# Resuscitated Sudden Cardiac Arrest of a Neonate with Congenital LQT Syndrome-Associated Torsades de Pointes: A Case Report and Literature Review

**DOI:** 10.3390/jcdd9060184

**Published:** 2022-06-09

**Authors:** Yen-Teng Hsu, Pi-Chang Lee, Yu-Hsuan Chen, Shu-Jen Yeh, Ming-Ren Chen, Kung-Hong Hsu, Chung-I Chang, Wei-Ting Lai, Wei-Li Hung

**Affiliations:** 1 Department of Pediatric Cardiology, Mackay Children’s Hospital, Taipei 104217, Taiwan; mmh.6156@mmh.org.tw (Y.-T.H.); j724603@gmail.com (Y.-H.C.); sjyehntu@gmail.com (S.-J.Y.); mingren44@gmail.com (M.-R.C.); 2 Department of Medical Education, Taichung Veterans General Hospital, Taichung 40705, Taiwan; pichanglee@vghtc.gov.tw; 3 Department of Surgery, Division of Cardiovascular Surgery, Mackay Memorial Hospital, Taipei 104217, Taiwan; erythlet@gmail.com (K.-H.H.); joeychang0423@gmail.com (C.-I.C.); 4 Department of Pediatrics, Division of Pediatric Cardiology, Hung Chi Women and Children’s Hospital, Taoyuan 320675, Taiwan; waiting.lai@gmail.com

**Keywords:** sudden infant death syndrome, long QT syndrome, torsades de pointes, implantable cardioverter defibrillator

## Abstract

Sudden infant death syndrome (SIDS), the most common cause of infant death in developed countries, is attributed to diverse trigger factors. Malignant cardiac dysrhythmias are potentially treatable etiologies, and congenital long QT syndrome (LQTS) is the most common cardiac ionic channelopathy confronted. β-Blockers or class Ib agents are the drugs of choice for the control of arrhythmias, and an implantable cardioverter defibrillator (ICD) should be considered for secondary prevention in survivors of lethal cardiac death. We report the case of a 4-day old neonate, later genetically confirmed as LQT type 3 (LQT3), who survived a pulseless torsades de pointes (TdP) attack and was successfully treated with propranolol, mexiletine, and ICD implantation.

## 1. Introduction

Sudden infant death syndrome (SIDS), the most common cause of infant death in developed countries, is a term that has been used to refer to a pathological entity for unexplained infant death since 1969. It is caused by various etiologies; among them, cardiac ionic channelopathies are potentially preventable and treatable. About 5% to 9.5% of SIDS cases may carry functionally significant genetic variants in long QT syndrome (LQTS) genes [1,2], mostly in *SCN5A* [2]. With the aid of genotype recognition, early diagnosis and timely treatments are possible. The most common initial dysrhythmic presentation of the LQTS affecting neonates is either sustained bradycardia or a 2:1 atrioventricular (AV) block [3,4]. Nevertheless, the neonatal onset of malignant ventricular tachyarrhythmia is quite rare [5]. Implantable cardioverter defibrillator (ICD) implantation is a class I recommendation for LQTS patients who have survived a cardiac arrest [6,7]. However, a standard technique for implanting an ICD in infants has not been established [8] due to the surgical difficulties caused by small body size, as well as the shortage of suitable infantile ICD leads and generators.

## 2. Case Report

A 2-day old female neonate born to a G2P2, 33-year-old mother was referred to our neonatal intensive care unit (NICU) due to dyspnea, cyanosis, and cardiac dysrhythmias. There was no remarkable antenatal history nor perinatal insult aside from fetal bradyarrhythmia recorded during fetal ultrasound examination in the second and third trimesters. The baby was born via vaginal delivery in a local hospital at 36 weeks gestational age with a birth weight of 3540 g (large for gestational age). The APGAR scores were 8 in the first minute and 9 in the fifth minute. Hours after birth, repeated cyanotic episodes occurred while feeding. Echocardiography detected decreased cardiac contractility (left-ventricular ejection fraction (LVEF), 35–40%), and pulmonary hypertension was suspected. Electrocardiography (ECG) showed variable QRS morphologies and durations, along with an extremely prolonged QTc interval (660 ms).

**Figure 1:**
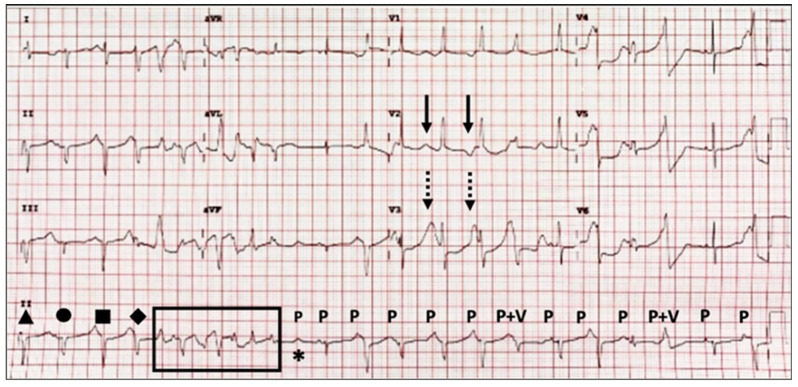
ECG on admission showing sinus rhythm with an extremely prolonged QTc interval of 623 ms under an HR of 127 bpm. The QRS morphology (triangle, circle, square, and diamond) and duration varied with T-wave alternans (arrows) and different T-wave morphologies (dotted arrows). The axis is northwest deviation. An AV block (asterisk), fusion beats (P + V), and non-sustained TdP (window) can also be observed. P, p wave; V, PVC.

**Figure 2:**
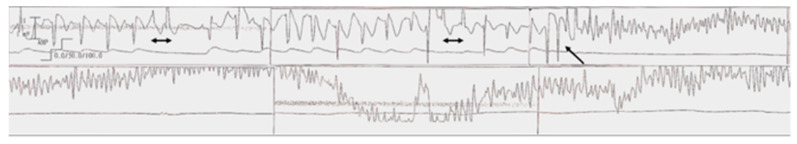
ECG rhythm strip on fourth postnatal day showing two PVC couplets (double arrow), followed by an R-on-T event (arrow) that initiated the TdP.

**Figure 3:**
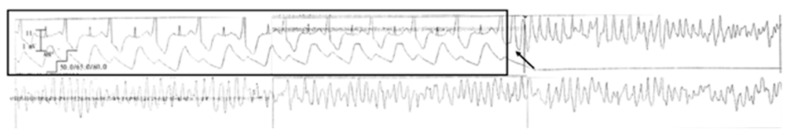
ECG rhythm strip on fifth postnatal day showing bigeminy PVC (window), followed by an R-on-T event (arrow) that initiated the TdP.

**Figure 4:**
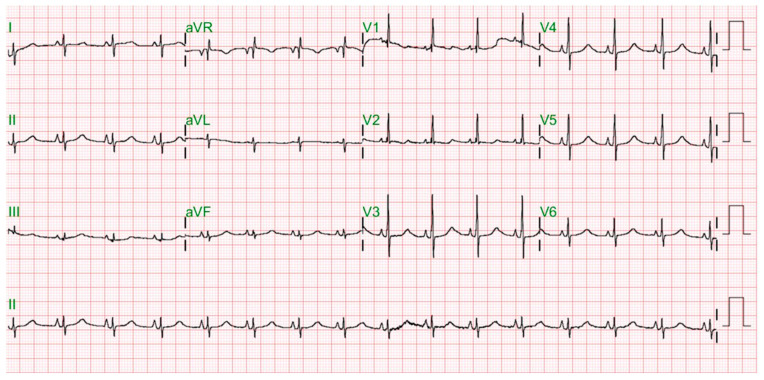
ECG after propranolol and mexiletine treatment showing normal sinus rhythm with a QTc interval of 400 ms under an HR of 92 bpm.

After the baby was admitted to the NICU, the 12-lead ECG screening disclosed a sinus rhythm with an extremely prolonged QTc interval of 623 ms under a heart rate (HR) of 127 beats per minutes (bpm). Both QRS morphology and duration varied, with T-wave alternans and different T-wave morphologies. A non-sustained torsades de pointes (TdP) was observed (Figure 1). Biochemistry tests, including cardiac enzymes (CK, CKMB, and Troponin-I) and serum electrolytes (Na, K, free Ca, Cl, and Mg), showed normal results. Two episodes of sustained TdP developed on her fourth and fifth postnatal days, which were converted to a sinus rhythm by cardiac defibrillation. Both TdP attacks were triggered by a preceding R-on-T event during frequent premature ventricular contractions (PVCs) (Figure 2 and Figure 3). An intravenous bolus of lidocaine (30 µg/kg) followed by continuous infusion (30 µg/kg/h) was given at first, while propranolol (2 mg/kg/day) and mexiletine (12 mg/kg/day) were prescribed with the suspicion of LQT3. She became drowsy, with some involuntary movements observed 3 days after lidocaine usage, and a reduced LVEF was also noticed. Lidocaine infusion was discontinued, and her consciousness and LVEF both recovered. The diagnosis of LQT3 was later confirmed by whole-exome sequencing of the girl’s peripheral blood genomic DNA, which identified a heterozygous missense variant in the SCN5A gene (c.5287G > A). Both parents’ genetic testing results were negative for such a mutation, suggestive of a sporadic *de novo* mutation. Her family members also received ECG screening, and their QTc intervals were normal. Her QTc interval normalized (Figure 4) after medical treatments, and no further TdP attack was observed.

**Figure 5:**
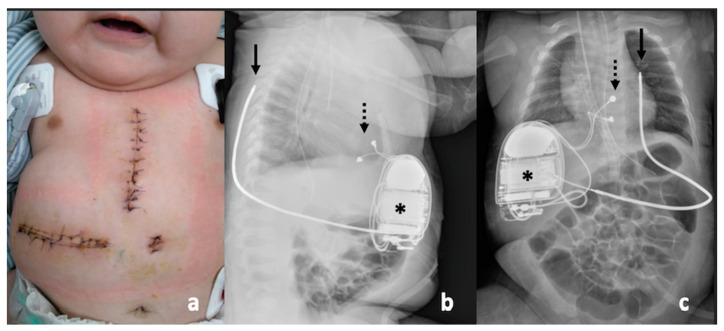
(**a**) Photo showing the postoperative wound after ICD implantation. (**b**) Chest X-ray, lateral view. The generator (asterisk) was implanted in a pocket in the right posterior rectus sheath. The subcutaneous defibrillator coil was located in the left subscapular position (arrow). The bipolar epicardial pacing leads (dotted arrow) were placed on the surface of the right ventricle. (**c**) Chest X-ray, anteroposterior view.

For patients who have survived a cardiac arrest, implantable cardioverter defibrillator (ICD) implantation is indicated (class 1 recommendation). ICD implantation was performed at 4 months of age, after her body weight reached 7 kg. Transvenous ICD implantation was unsuitable due to her small body size; therefore, we chose the DF-1 ICD (Evera XT DR; Medtronic, Tolochenaz, Switzerland), the subcutaneous defibrillator coil (6996SQ; Medtronic), and the bipolar epicardial pacing leads (4968; Medtronic) for surgical implantation. The generator was implanted in the right posterior rectus sheath, the subcutaneous defibrillator coil was located in the left subscapular position, and the bipolar epicardial pacing leads were inserted on the surface of the right ventricle (Figure 5a–c). The generator and the coil were placed on opposite sides of the heart, allowing the electric current of defibrillation to flow between them. A programmed delivery of 15 J of DC shock successfully reverted to a sinus rhythm in the defibrillation threshold testing (DFT). No adverse event or malfunction of the device occurred following discharge, and the latest chest film showed proper positions of the ICD, the pacing leads, and the subcutaneous coil. Remote monitoring revealed no recurrence of TdP or any ICD shock under medical control with mexiletine (12 mg/kg/day) and propranolol (3 mg/kg/day). Her growth and development have been appropriate for her age during the 6-month follow-up period.

## 3. Discussion

### 3.1. Linkage of SIDS and LQTS

The incidence of SIDS is estimated between 0.4 and 0.5 per 1000 live births in Western countries, with peak incidence occurring between 2 and 4 months of age with male predominance [9]. Previous studies have reported that a prolonged QT interval in the first week of life represents an important risk factor for a lethal event [10]. About 5% to 9.5% of SIDS cases carry functionally significant LQTS genetic variants [1,2], mostly in *SCN5A* (50%), followed by *KCNQ1* (19%), *KCNH2* (19%), *CAV3* (11%), and *KCNE2* (4%) [2]. Among all confirmed LQTS genetic variants, most sporadic mutations identified belong to the *SCN5A* gene. Sporadic mutations in the cardiac ion channel genes may explain why the ECGs of parents and siblings of SIDS victims do not demonstrate QT interval prolongation [10].

### 3.2. Presentation of LQTS in Infancy

LQTS should be considered in fetuses with a prenatal diagnosis of bradycardia, hydrops, or complex arrhythmias [3]. Neonates with a history of significant fetal bradycardia (fetal HR ≤ 110 bpm or third percentile for a gestational age-matched cohort) should receive ECG evaluation soon after delivery [11]. The most common postnatal ECG findings of LQTS include bradycardia, bradyarrhythmia (mostly 2:1 AV block), ventricular arrhythmia, or TdP [3,4,12,13]. The observed 2:1 AV block was most likely functional (“pseudo” AV block) because these P waves are inscribed within the T wave, and therefore likely occurred during the refractory period of the ventricles [14].

The varying QRS morphology and duration in our case may be attributed to impaired AV conduction in LQTS, as also described in previous reports [14,15]. Those aberrancies could be followed by different T-wave morphologies, increasing the difficulty of identifying the type of LQTS; as known, LQT1, LQT2, and LQT3 have broad-based, flattened, and late-onset T-waves, respectively [6].

In an infantile ECG screening showing QT prolongation, it is important to weigh the balance between false positives and false negatives when choosing appropriate timing and cutoff values. Cutoff values of QTc ≥470 ms or ≥460 ms have been proposed in previous studies [16,17,18]. Since QTc changes physiologically with postnatal age, and considering the various risk factors in screened cases, a single timing and a single cutoff value may not apply to all circumstances when aiming to detect SIDS-vulnerable infants.

### 3.3. High-Risk Factors for Sudden Cardiac Death Due to LQTS in Infancy

Infants with QTc > 600 ms are at high risk for sudden death. Macroscopic TWA is a marker for high cardiac electrical instability and is considered an important warning signal for imminent TdP (high specificity, low sensitivity) [19]. When an AV block is present in congenital LQTS patients, particularly among neonates, the likelihood of developing a cardiac event increases significantly and is associated with poor prognosis [4]. Other type-specific high-risk factors include QTc ≥ 500 ms with LQT1, females with the LQT2 genotype, and males with the LQT3 genotype [7,20,21].

### 3.4. Medical Treatment of LQTS in Infancy

Our case had a normalized QTc interval and no further TdP attacks after applying propranolol and mexiletine. Sodium channel blockers (lidocaine, mexiletine, flecainide) are known to be specific treatments for LQT3 patients, with varying selectivity for sodium channels over the peak sodium current [22]. Recently, ranolazine, a more specific sodium channel inhibitor, has been tested.

The use of β-blocker therapy in LQT3 patients remains controversial. The prescription of β-blockers is well justified for its prevention of sympathetic nervous system excitation, which can subsequently trigger cardiac events among LQTS patients. However, the trigger for cardiac events in LQT3 patients is less likely to be adrenergic, instead appearing to be predominantly associated with bradycardia [23,24]. Nevertheless, a previous study reported an 83% reduction in cardiac events in LQT3 female patients taking β-blocker therapy, however the efficacy in male patients was not conclusive [25].

The antiarrhythmic effect of β-blockers is attributed to the prevention of early afterdepolarizations by blocking the adrenergic-driven boost in calcium currents. Only propranolol reduces the QTc interval to some extent by blocking the late inward current of sodium [26]. The nonselective β-blockers (nadolol and propranolol) have been advocated as the most effective drugs [27,28], but only the liquid form of propranolol is available for infancy. On the other hand, metoprolol and atenolol are less effective and should be avoided, at least in symptomatic patients [6].

### 3.5. ICD Implantation in Infancy

In addition to medical treatment, cardiac pacing to prevent bradycardia and ICD implantation have been advised in LQT patients who have survived a cardiac arrest or in patients at anticipated high risk for life-threatening cardiac arrhythmias [6,29].

However, a standard implantation technique for children, especially infants, has not yet been established, mainly because the ICD system faces space constraints, resulting in a higher rate of postoperative complications [30]. Different ICD approaches, including pericardial, epicardial, and subcutaneous systems, are all potential choices [31].

Zahedivash et al. [32] recently reported a 15-case experience of implantation by epicardial attempts with the devices of ICD (Evera, Protecta, or Cobalt; Medtronic), epicardial leads (4968 CapSure Epi sensing leads; Medtronic), and pericardial coils (6937A Transvene CS ICD coil; Medtronic) in their retrospective study from 2009 to 2021. They generally put the epicardial leads on the RA or RV apex, respectively. The short, 50 mm ICD coils (6937A; Medtronic) were placed in the left posterior pericardial space. The generators were fixed at the right upper abdomen. The body weight range of their patients was from 2.5 kg to 18.0 kg. This approach revealed good short term results, with a 20% appropriate shock rate and no inappropriate shock during follow-up. The immediate postoperative complications included chylothorax in one patient and three episodes of feeding intolerance.

Compared to epicardial or pericardial systems, subcutaneous systems certainly seem to be superior in avoiding constrictive pericarditis or even cardiac tamponade [30]. However, an elevated defibrillation threshold and migration or breakage of the leads are of concern [33,34]. The DFT in Zahedivash’s study for the epicardial system was only 0.90 (IQR, 0.68–1.04) J/kg. The subcutaneous systems were found to have a significantly higher defibrillation threshold compared to the epicardial systems [8].

In our case, we chose the DF-1 ICD (Evera XT DR; Medtronic, Tolochenaz, Switzerland), the subcutaneous defibrillator coil (6996SQ; Medtronic), and the bipolar epicardial pacing leads (4968; Medtronic) for several reasons: (1) The body size was not suitable for transvenous access. (2) Back-up pacing was still required since we wanted to optimize the β-blocker therapy. (3) The shortage of short-length trans-venous DF1 defibrillator coils (the aforementioned Medtronic 6937A Transvene CS ICD coils are not available in Taiwan).

Treatment should be individualized on the basis of true patient-oriented needs. Further investigation is warranted in order to clarify the outcomes and specific complications of the different systems, especially in infants [31].
